# Predictive machine learning approaches for the microstructural behavior of multiphase zirconium alloys

**DOI:** 10.1038/s41598-023-32582-9

**Published:** 2023-04-03

**Authors:** Tamir Hasan, Laurent Capolungo, Mohammed A. Zikry

**Affiliations:** 1 North Carolina State University, Raleigh, USA; 2 Los Alamos National Laboratory, Los Alamos, USA

**Keywords:** Materials science, Structural materials, Metals and alloys

## Abstract

Zirconium alloys are widely used in harsh environments characterized by high temperatures, corrosivity, and radiation exposure. These alloys, which have a hexagonal closed packed (h.c.p.) structure thermo-mechanically degrade, when exposed to severe operating environments due to hydride formation. These hydrides have a different crystalline structure, than the matrix, which results in a multiphase alloy. To accurately model these materials at the relevant physical scale, it is necessary to fully characterize them based on a microstructural fingerprint, which is defined here as a combination of features that include hydride geometry, parent and hydride texture and crystalline structure of these multiphase alloys. Hence, this investigation will develop a reduced order modeling approach, where this microstructural fingerprint is used to predict critical fracture stress levels that are physically consistent with microstructural deformation and fracture modes. Machine Learning (ML) methodologies based on Gaussian Process Regression, random forests, and multilayer perceptrons (MLP) were used to predict material fracture critical stress states. MLPs, or neural networks, had the highest accuracy on held-out test sets across three predetermined strain levels of interest. Hydride orientation, grain orientation or texture, and hydride volume fraction had the greatest effect on critical fracture stress levels and had partial dependencies that were highly significant, and in comparison hydride length and hydride spacing have less effects on fracture stresses. Furthermore, these models were also used accurately predicted material response to nominal applied strains as a function of the microstructural fingerprint.

## Introduction

Zirconium alloys are widely used in settings where high temperature resistance, corrosion resistance, or low susceptibility to radiation are required^1^. They can be used as cladding for uranium in nuclear reactors, where exposure to high temperature heavy water can cause defects within the microstructure caused by hydrogen accumulation^2,3^. These defects have been shown to degrade the mechanical behavior properties of zirconium alloys, such as ultimate tensile stress, ductility, and fracture strains^4,5^. These microstructural characteristics can play a critical role in material performance during long-term storage and in incidents, such as loss of coolant accidents (LOCA)^6^. It is, therefore, essential to understand and predict the impact of hydrides on these materials.

Experimental studies of hydrided zirconium materials have indicated that hydrided materials, along with the geometry associated with the hydrides, are instrumental in characterizing the material response. For hydride formation occurring during delayed hydride cracking (DHC), Shi and Puls concluded that the size and shape of the hydrides precipitated at the crack tip negatively affected the stress intensity factor, and thereby crack propagation^7^. The fracture toughness in Zircaloy-4 sheet has been experimentally shown to decrease as hydrogen content increased, and as the proportion of radially oriented hydrides increased^8^. Higher temperatures were found to reduce crack propagation due to increased ductility. Studies of fracture in hydrided materials have shown that hydrides tend to cause brittle failure at temperatures below 100 °C, with the matrix exhibiting ductile failure^9^. Colas et al. studied the thermal dependence of hydride formation further and quantified the elastic strains due to the formation of different hydride orientations^10^. Sharma et al. found that fracture toughness was reduced with the formation of hydrides, and even more so for radial hydride formation, with approximately an 80% reduction when compared with circumferential hydrides^11^. Studies of fatigue in hydrided zirconium alloys have also shown a strong preference for microcrack formation in radially oriented hydrides^12^.

Computational analyses of hydrided materials have been performed at various length scales, with phase field modeling (PPM) and density functional theory (DFT) to study hydride formation^13^, reorientation^14^, embrittlement^15^, and their effects on crack propagation. At larger length scales, finite element method (FEM) models are used from microstructural to the macro. Liu et al. examined a component level model informed by grain-scale simulations of hydrides and found that a linking effect between individual hydrides influenced fracture crack paths and material failure^16^. Therefore, it is necessary to examine populations of hydrides to fully understand how larger scale systems are affected.

While these investigations have provided a viable lens by which to understand hydrided zirconium alloys, there has not yet been a unified model that relates how failure nucleates and evolves. Furthermore, a validated statistically based or ML ranking of the parameters known to be important has not yet been attained. A major drawback to both experimental studies and the modeling of hydrided zirconium materials is the time and lack of capability to validate predictions for nonlinear behavior.

Various studies have proposed the use of reduced order models (ROMs) to characterize materials^17,18^. These ROMs are not computationally expensive to query and can provide a statistically significant representation of the data they were trained on. These approaches, however, lack a sufficient expression for the spatial extreme phenomena that lead to material failure and crack propagation, and there are no such models that directly address the question of hydrided zirconium. Hence, a ROM predicting thermo-mechanical fracture behavior for a high dimensional representation of the hydrided zirconium matrix is critical for understanding the characteristics of fracture in these materials. It is also essential for the large-scale modeling of these materials by reducing the computational burden of fundamentally understanding high fidelity materials at a macro scale.

**Figure 1:**
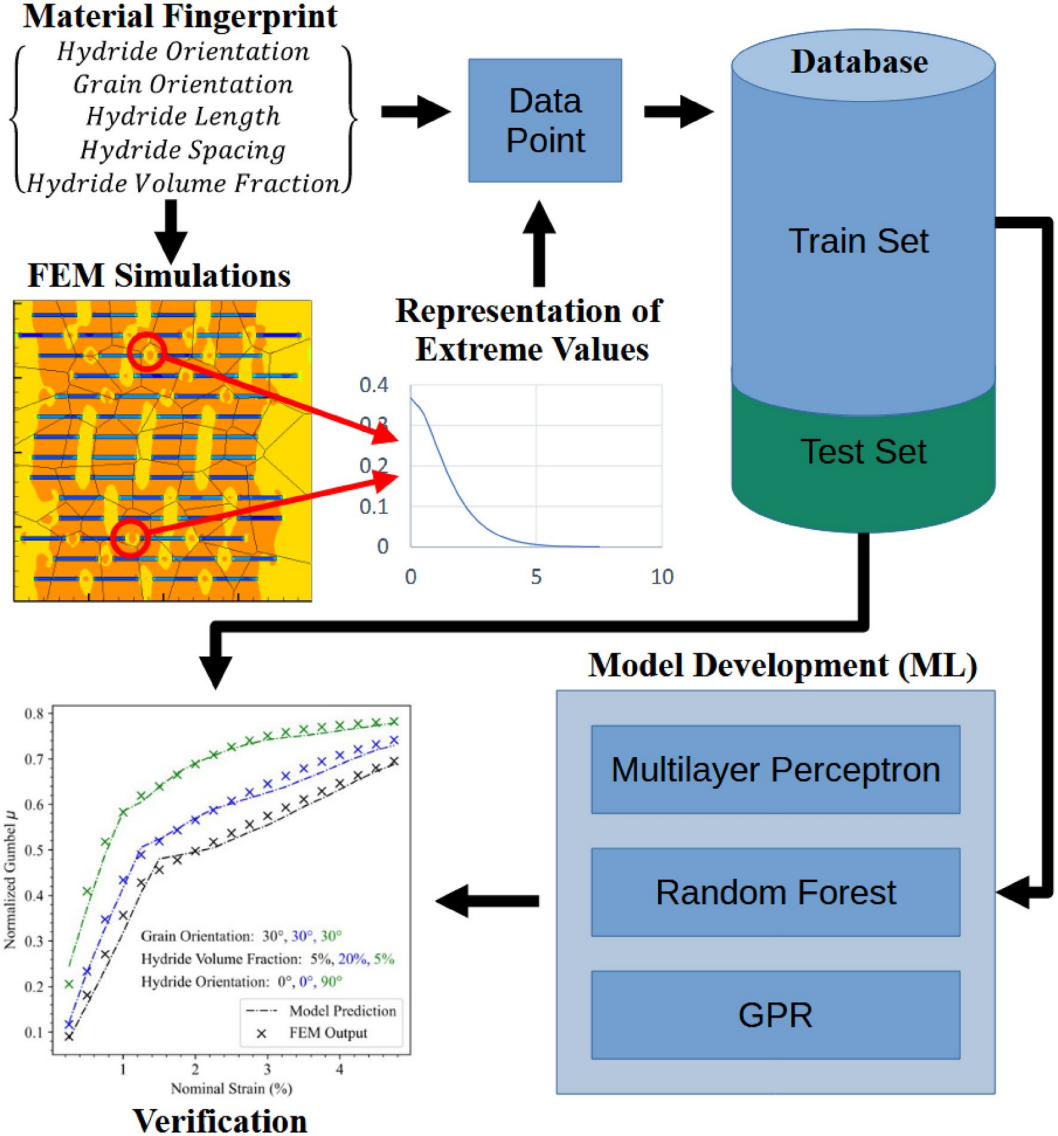
An outline of the proposed ML modeling process**.** The FEM results are distilled into a representation of their extrema, and then various ML approaches were tested to determine which was best suited to model the data. These models have the material fingerprint as inputs, and then output the material state for a given level of strain.

Therefore, to address these shortcomings, a dislocation density-based crystalline plasticity approach is used to model a representative solution space of the hydrided zirconium problem, which provides a database that can be interrogated by ML approaches to understand and predict how microstructural behavior affects fracture nucleation and propagation in hydrided alloys. The microstructural material fingerprint includes face input categories: hydride orientation, grain orientation, hydride volume fraction, hydride length, and hydride spacing. These fingerprint components ensure that the database and resulting models can be queried using new findings that are obtained experimentally. This is an important aspect of a computational materials database because it creates the link between simulated and experimental data and provides added context to experimental data that would be difficult to measure manually^19^. The database was used to generate statistically significant models of critical fracture stress in the five-dimensional space that describes the material fingerprint. An extreme value theory (EVT) framework for reducing the dimensionality of a meshed model’s output was used to create a heavy-tailed representation of the material dynamics such that the resulting models best describe extreme fracture stress states. Furthermore, these models were interrogated to determine the relative importance of each parameter comprising the material fingerprint. The process by which the raw data is processed via EVT, fit using various model types, and finally used to generate a prediction of the extremes of the material critical fracture stress state given a material fingerprint and strain level, is shown in Fig. 1.

## Methods

### Multiple-slip crystal plasticity dislocation-density

A dislocation-density crystalline plasticity approach was used in conjunction with FEM to generate the database used in this study. The crystalline plasticity approach was developed by Zikry and Kao^20^ and Shanthraj and Zikry^21^ and uses a set of partial differential equations to describe the dislocation evolution within a unit area called dislocation density. Separate equations are used for mobile and immobile dislocation densities, ${\rho }_{m}$ and ${\rho }_{im}$, and a set of nondimensional coefficients are used to describe the sourcing, trapping, annihilation, immobilization, and recovery of dislocations^21^. The dislocation density evolution equations are

1 $$\frac{d{\rho }_{m}^{\alpha }}{dt}=\left|{\dot{\gamma }}^{\alpha }\right|\left(\frac{{g}_{sour}^{\alpha }}{{b}^{2}}\left(\frac{{\rho }_{im}^{\alpha }}{{\rho }_{m}^{\alpha }}\right)-{g}_{mnter-}^{\alpha }-{\rho }_{m}^{\alpha }-\frac{{g}_{immob-}^{\alpha }}{b}\sqrt{{\rho }_{im}^{\alpha }}\right),$$

2 $$\frac{d{\rho }_{im}^{\alpha }}{dt}=\left|{\dot{\gamma }}^{\alpha }\right|\left({g}_{mnter+}^{\alpha }{\rho }_{m}^{\alpha }+\frac{{g}_{immob+}^{\alpha }}{b}\sqrt{{\rho }_{im}^{\alpha }}-{g}_{recov}{\rho }_{im}^{\alpha }\right),$$

where *g*_*sour*_ is the coefficient pertaining to an increase in the mobile dislocation density due to dislocation sources, *g*_*mnter*_ is the coefficient related to the trapping of mobile dislocations due to forest intersections, cross slip around obstacles, or dislocation interactions, *g*_*recov*_ is a coefficient related to the rearrangement and annihilation of immobile dislocations, and *g*_*immob*_ is related to the immobilization of mobile dislocations. These coefficients, which have been nondimensionalized, are summarized in Table 1, where ${f}_{0}$*,* and $\varphi$ are geometric parameters. *H*_*0*_ is the reference activation enthalpy, l_c_ is the mean free path of a gliding dislocation, b is the magnitude of the Burgers vector, and *ρ*_*s*_ is the saturation density. It should be noted that these coefficients are functions of the immobile and mobile densities, and hence are updated as a function of the deformation mode. Shear slip rate, $\dot{\gamma }$, is a measure of the accumulated plastic strain on a material that is related to the mobile dislocation activity in a material as

3 $${\dot{\gamma }}^{(\alpha )}={\rho }_{m}^{(\alpha )}{b}^{\left(\alpha \right)}{v}^{\left(\alpha \right)},$$

where ${v}^{(\alpha )}$ is the average velocity of mobile dislocations on slip system $\alpha$.

**Table 1:** Coefficients for dislocation-density equations (Eqs. 1–2).

Coefficients	Expression
$g_{sour}^{\alpha }$	$b^{\alpha } \varphi \sum\limits_{\beta } {\rho_{im}^{\beta } }$
$g_{mnter - }^{\alpha }$	$l_{c} f_{0} \sum\limits_{\beta } {\alpha_{\alpha \beta } } \left[ {\frac{{\rho_{m}^{\beta } }}{{\rho_{m}^{\alpha } b^{\alpha } }} + \frac{{\dot{\gamma }^{\beta } }}{{\dot{\gamma }^{\alpha } b^{\beta } }}} \right]$
$g_{immob - }^{\alpha }$	$\frac{{l_{c} f_{0} }}{{\sqrt {\rho_{im}^{\alpha } } }}\sum\limits_{\beta } {\sqrt {\alpha_{\alpha \beta } } } \rho_{im}^{\beta }$
$g_{mnter + }^{\alpha }$	$\frac{{l_{c} f_{0} }}{{\dot{\gamma }^{\alpha } \rho_{m}^{\alpha } }}\sum\limits_{\beta ,\gamma } {n_{\alpha }^{\beta \gamma } \sqrt {\alpha_{\beta \gamma } } } \left[ {\frac{{\rho_{m}^{\gamma } \dot{\gamma }^{\beta } }}{{b^{\beta } }} + \frac{{\rho_{m}^{\beta } \dot{\gamma }^{\gamma } }}{{b^{\gamma } }}} \right]$
$g_{immob + }^{\alpha }$	$\frac{{l_{c} f_{0} }}{{\dot{\gamma }^{\alpha } \sqrt {\rho_{im}^{\alpha } } }}\sum\limits_{\beta } {n_{\alpha }^{\beta \gamma } \sqrt {\alpha_{\beta \gamma } } } \rho_{im}^{\gamma } \dot{\gamma }^{\beta }$
$g_{{re{\text{cov}} }}^{\alpha }$	$\frac{{l_{c} f_{0} }}{{\dot{\gamma }^{\alpha } }}\left( {\sum\limits_{\beta } {\sqrt {\alpha_{\alpha \beta } } } \frac{{\dot{\gamma }^{\beta } }}{{b^{\beta } }}} \right)e^{{\left( {\frac{{ - H_{0} \left( {1 - \sqrt {\frac{{\rho_{im}^{\alpha } }}{{\rho_{s} }}} } \right)}}{kT}} \right)}}$

The orientation relationships (ORs) between the $\delta$ hydrides examined in this work and the surrounding matrix were developed in Mohamed and Zikry^22^ as

4 $${\left(0001\right)}_{hcp}// {\left(111\right)}_{fcc} and {\left[11\overline{2 }1\right]}_{hcp}//{\left(\overline{1 }10\right)}_{fcc},$$

and represent the plane relationships between the h.c.p. material and the f.c.c. material.

#### Finite-element Input categories and model

Each simulation was developed according to its own material fingerprint and consisted of a plane strain model with displacement control for the FE model. The average mesh size was 60,000 elements and consisted of 49 zirconium h.c.p. alloy grains and approximately 50 f.c.c. hydrides, for conditions including hydride spacing and hydride length based on the fingerprint. Strain was applied uniaxially, and grain orientation with respect to the loading axis was defined using the value of the material fingerprint parameter. The strain rate was constant at 10 ${s}^{-1}$. Grain orientations were defined as the angle that the zirconium alloy’s [0 0 1 0] axis forms with respect to the loading axis, which is uniaxial at the [0 0 1 0] global direction. Changes to this parameter rotated the grain with respect to the [0 1 0 0] normal axis. Mohamed and Zikry^23^ validated the material properties used in the simulations, which are presented in Table 2.

**Table 2:** Material properties.

Property	Zircaloy-4	$\delta$*-*hydrides
Young’s modulus (GPa)	35	132
Static yield stress (MPa)	220	220
Poisson’s ratio	0.349	
Rate sensitivity coefficient	45	
Initial immobile dislocation density (m^−2^)	1 × 10^10^	
Initial mobile dislocation density (m^−2^)	1 × 10^7^	
Burger’s vector	1 × 10^–10^	
Fracture stress (MPa) (N/mm^2^)	700	1000
Final applied tensile nominal strain (%)	12	12

#### Database generation

To characterize the solution space of all possible material fingerprints, a total of 210 simulations were simulated using FEM. The material fingerprints for each simulation consisted of five material parameters and were chosen according to uniform distributions bounded by the values in Table 3. The parameter values were chosen from a grid of 4 equally spaced values from within these bounds. In addition to modifying grain orientation according to the range within these bounds, grain to grain misorientation was randomized at a maximum of 10°. These parameters influence dislocation activity and fracture, and the specific values used correspond to experimental values^4,11,23,24^. To sample from the solution space, the trajectory method used in the Elementary Effects method (implemented in SAlib) was used^25,26^.

**Table 3:** Hydride microstructural distributions.

Material fingerprint	Minimum value	Maximum value
Hydride Length	0.5E−5 m	3.0E−5 m
Parent Grain Orientation	0°	90°
Hydride Volume Fraction	5%	20%
Hydride Orientation	0°	90°
Hydride Spacing	2.5E−6 m	1.0E−5 m

#### Model development

To avoid numerical issues with the model input parameters being at different physical scales, the parameters were processed by centering the mean of each feature around zero and scaling to unit variance using the StandardScaler function in Scikit-Learn (Version 0.23.2)^27^. The critical fracture stress values were also scaled by 100 MPa.

When training the models, the data was randomly split into an 85% training set and a 15% validation set. The training set was used to train model hyperparameters. The hyperparameters were randomly chosen for training within predefined ranges chosen for each model type. Typically, 50,000 iterations within the hyperparameter space were used along with a fivefold cross validation technique to reduce overfitting to the data. After the best possible model hyperparameters were found, the model was tested on the validation set which comprised 15% of the original data, and which had not been used to train the model. The goodness of fit, for this model, is the measurement of the model’s performance in predicting this validation set.

A linear regression using OLS was used to provide a benchmark for the other modeling methods explored in this work. Because the simulations are non-linear, there was only a small likelihood of attaining a high level of accuracy with this class of models. However, linear regression provides the most interpretable model output of any of the other modeling systems. Scikit-Learn’s LinearRegression function was used to produce these models^27^.

The random forest regression model was used to generate a model. It is comprised of an ensemble of decision trees whose outputs are averaged. The result is a general model that provides accurate predictions in high dimensional spaces^28^. Decision tree-based methods are also helpful because their output can be interrogated, though it may be cumbersome to do so for an ensemble of decision trees. 100 estimators were used for each regression model, corresponding to 100 decision trees, which would make this kind of interpretation difficult. Other methods exist to interpret the output of a random forest regressor, and they are implemented here. The importance of each input parameter can also be determined using methods such as recursive feature elimination. Scikit-Learn’s RandomForestRegressor^27^ was used.

A multilayer perceptron, or neural network, was also fitted to the data. While neural networks are the least interpretable method presented in this study, they have also been shown to be powerful estimators for highly dimensional data. The models presented here were comprised of 3 hidden layers with 5 neurons each. Scikit-Learn’s MLPRegressor function was used to train and test these estimators^27^.

Gaussian Process Regression (GPR) was chosen as a model type because of its built-in measure of uncertainty. A combination kernel comprised of a Matern kernel and an Exponential Sine Squared kernel were used for training. The sinusoidal attribute of this kernel was a result of the prior understanding that material properties tend to follow a sinusoidal path as a non-isotropic material is rotated. The Matern kernel additionally allowed the model to effectively capture discontinuities in the solution space. The GaussianProcessRegressor function within the SciKit-Learn package was used to train the models, and a cross validated randomized search was used to find length scale, the Matern $\nu$ parameter, and the periodicity parameter for the exponential sine squared kernel^27^.

#### Application to fracture probability

The purpose of this study is to obtain ROMs that describe the fracture stress state of a material given its material fingerprint and strain level. This is performed by predicting the $\mu$ value of a Gumbel distribution trained to the 95th percentile of each data set. These $\mu$ values are normalized by the fracture stress to provide physically based insights. These models can provide critical microstructural fracture predictions without FEM models or experimental measurements, and is a representation of incipient fracture within the material. The fracture critical stress information predicted from these models can then be used in conjunction with other computational and experimental methods to determine the likelihood of failure. These predictions are the link between the material fingerprint and the fracture probability for that material at a certain strain level.

## Results and discussion

**Figure 2:**
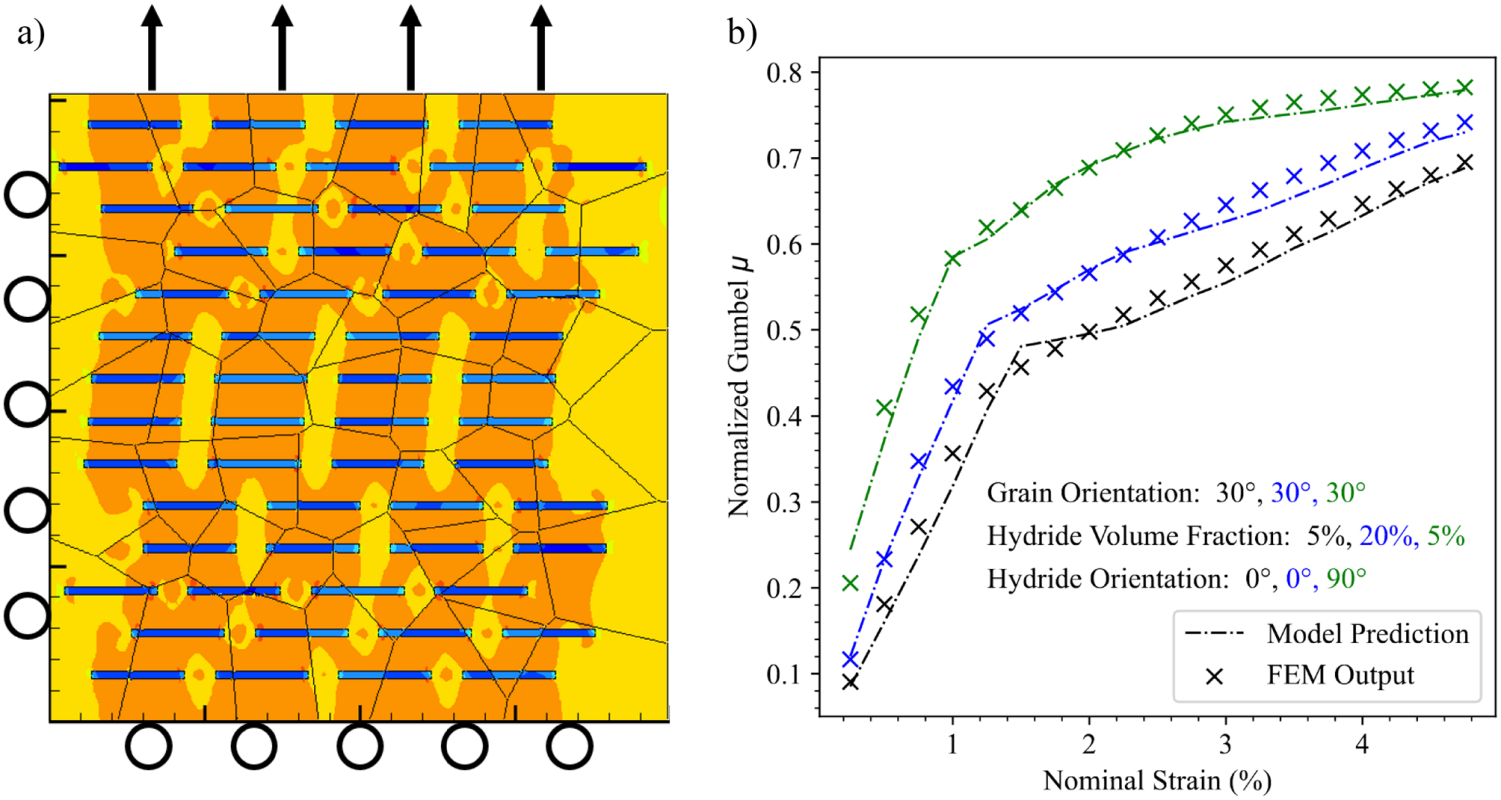
(**a**) The finite-element representation of a multiphase hydrided zirconium alloy representative volume element. The arrows correspond to the applied tensile displacements. (**b**) Model verification as a function of strain response. The coloring indicates a subset of material parameters as referenced in the center legend. Grain orientation, hydride volume fraction, and hydride orientation were varied and the model prediction at various strain levels was compared to FEM output.

The FE models were obtained from crystal plasticity simulations for the hydrided zirconium alloy. The database was populated by randomly modifying five parameters of interest using four predefined levels between a minimum value and a maximum value. These parameters are given in Table 3. A representative FE mesh for quasi-static plane strain loading conditions that correspond to a strain-rate of 0.01/s is shown in Fig. 2a. The nonlinear FE approach is based on the methodology detailed in^22,23^. In addition to the mechanical loading conditions, there are thermal boundary conditions of 20 °C applied on all four sides of the mesh. As discussed in^22,23^, the thermal changes are, however minimal due to the applied quasi-static loading rate and that the reference stress on each slip system is also thermally independent. A 0° reference angle is defined with respect to the loading direction. The grain orientation parameter is set to 0° when the zirconium alloy parent material [0 0 1 0] direction is coincident with the loading direction. Changes to the parent grain orientation are achieved by rotating the parent material about its [0 1 0 0] axis. This allows us to quantify the effect of different grain orientations with respect to the loading axis according to a single angle parameter. Furthermore the FE model was validated with predictions pertaining to zirconium alloys as detailed in^22,23^^.^

**Figure 3:**
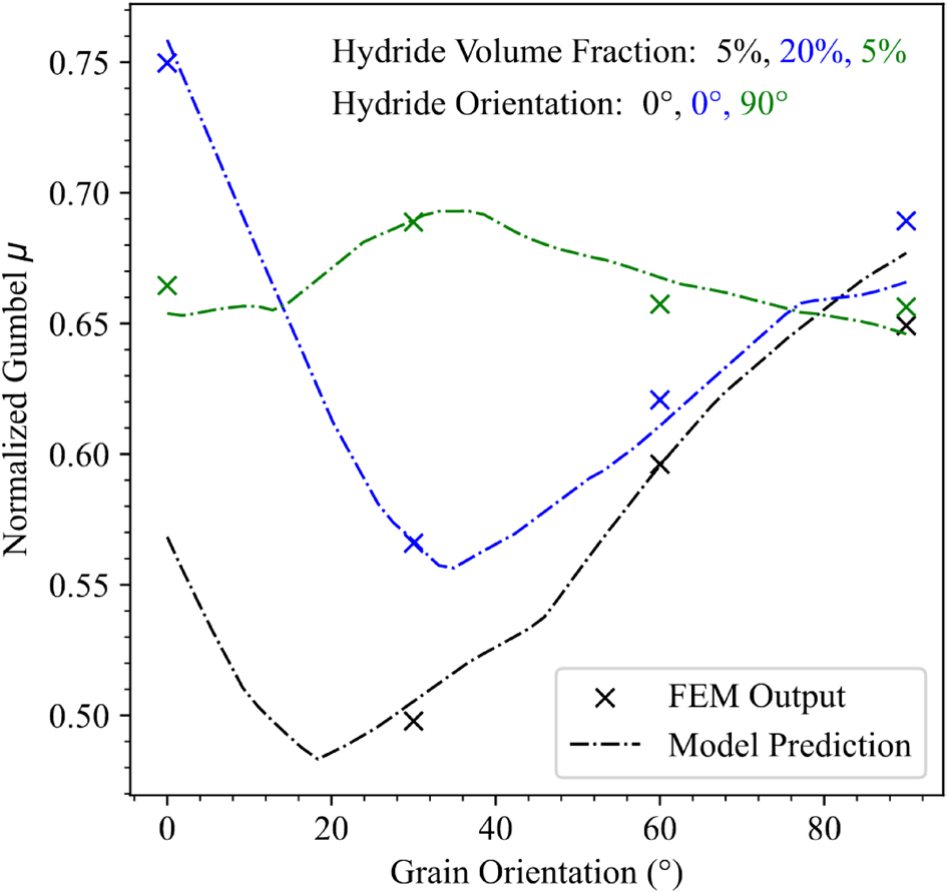
Model verification as a functions of grain orientation**.** The colors denote a subset of material parameters as referenced in the legend in the top right. Variations in hydride volume fraction and hydride orientation are also included. FEM output data is not shown for the 5% HVF, 0° hydride orientation, 0° grain orientation case because that case was not part of the training dataset.

The four ML modeling methods were fit to the training data using a cross validated technique and randomization to tune hyperparameters in all cases. The coefficient of determination values resulting from testing the models against the validation set are shown in Table 4. The coefficient of determination, or r^2^, makes a comparison between the errors at each data point and the variation observed in the data points. A small ratio between the squared quantities indicates that the model error is less than the average variation in the data points, and results in an r^2^ value closer to 1. The ordinary least squares method is included as a benchmark and reasonable predictability was not expected owing to the nonlinear nature of the problem. The most robust results came from fitting neural networks to the data, but the GPR and Random Forest methods also provided excellent results. In addition to developing models specifically at individual strain levels, the MLP, GPR, and random forest methods were used to develop models, with strain as a dependent variable, in addition to the features of the material fingerprint. By incorporating nominal strain as an additional parameter, a six-dimensional input parameter space is obtained with more data points. The scale of the distribution was also included as a second output variable. The ${r}^{2}$ values for these models are given in Table 4. The values improved over those of individual strain levels, owing both to the large variation in material response during large increments in strain and the additional data available for training and testing. The model verifications were performed on a held-out test set. Figures 2b and 3 show comparisons between FEM database and predicted model values for selected material fingerprints and variations in both strain and grain orientation for the MLP. The predictions from the model provide a continuous representation of the solution, as opposed to the FEM data, which was developed using the Morris trajectory scheme, and therefore, only exists in predefined intervals within the parameter space.

**Table 4:** Validation set r^2^ values for critical fracture stress.

Model type	0.75% Strain	2.25% Strain	3.25% Strain	All Strains
GPR	0.87	0.61	0.85	0.98
Neural Network	0.86	0.88	0.93	0.98
Random Forest	0.86	0.80	0.88	0.46
Ordinary Least Squares	0.50	0.44	0.51	0.69

The material dependency on nominal strain level is already well established, the models trained to specific strain levels can then be used to examine the dominant microstructural fingerprint features in the following sections. The neural network-based models were used for this analysis because the ${r}^{2}$ values consistently higher than the GPRs, and the ability of the model to describe the solution space was determined to be more valuable than the measure of uncertainty that the GPRs provide.

### Feature elimination

Recursive feature elimination (RFE) was applied to determine which features can be eliminated from a given model without affecting the model’s predictive power. Features are randomly removed, and recursion is used to drive towards the smallest set of possible features. Fivefold cross validation was used to test the smaller feature sets to ensure that the model’s continued efficacy was not random. This method of feature tuning is only available for models that assign weights to features, and the random forest model was the only such type considered in this work. Applying RFE to the random forest models previously described at three strain levels resulted in the feature importance ranking as outlined in Table 5. Grain and hydride orientations are consistently the highest ranked features, followed by volume fraction. Hydride spacing is consistently the least important parameter. This is most likely because spacing between hydrides on the axis perpendicular to the hydrides was also controlled by the hydride volume fraction.

**Table 5:** Feature importance ranking for 3 random forest models.

Feature	1.25% Strain	1.5% Strain	2.75% Strain
Hydride length	3	3	2
Grain orientation	1	1	1
Hydride volume fraction	2	2	1
Hydride orientation	1	1	1
Hydride spacing	4	4	3

### Feature significance

**Figure 4:**
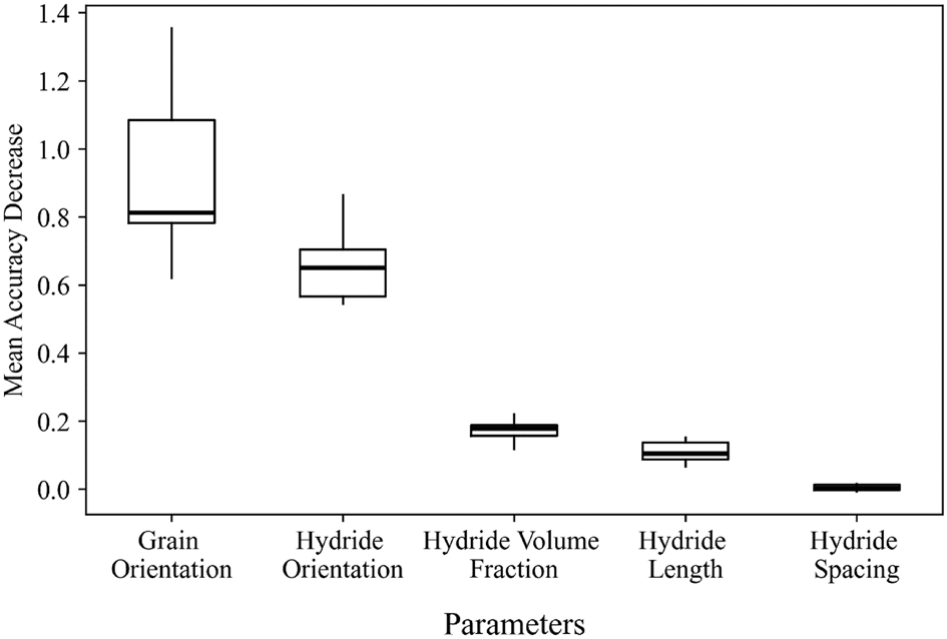
Permutation importance study**.** Performed for all parameters, calculated for the neural network trained for the 2.75% strain level.

To determine the importance of individual features, a permutation scheme was used. Each parameter was randomly permuted to determine the resulting impact on the model output, measured in the amount by which the coefficient of determination (${r}^{2}$) decreased when the model was scored against the training dataset with one parameter permuted. Each parameter was permuted a total of 10 times, and the results showing the impact on the coefficient of determination are shown as boxplots in Fig. 4. This was calculated for the neural network model trained at the 2.75% strain level, but similar plots produced for all strain levels indicated similar information, as did the random forest model set. These results correspond to the feature importance ranking for the 3 random forest models shown in Table 5. Hydride orientation and grain orientation have the highest impact on the model’s ability to explain the variation of critical fracture stress in the data. Hydride volume fraction, hydride length, and hydride spacing all had considerably reduced impact on the outcome, and the consistency of the reduced impact (tight range) indicated that an appropriate number of permutations were present to differentiate the less important parameters.

**Figure 5:**
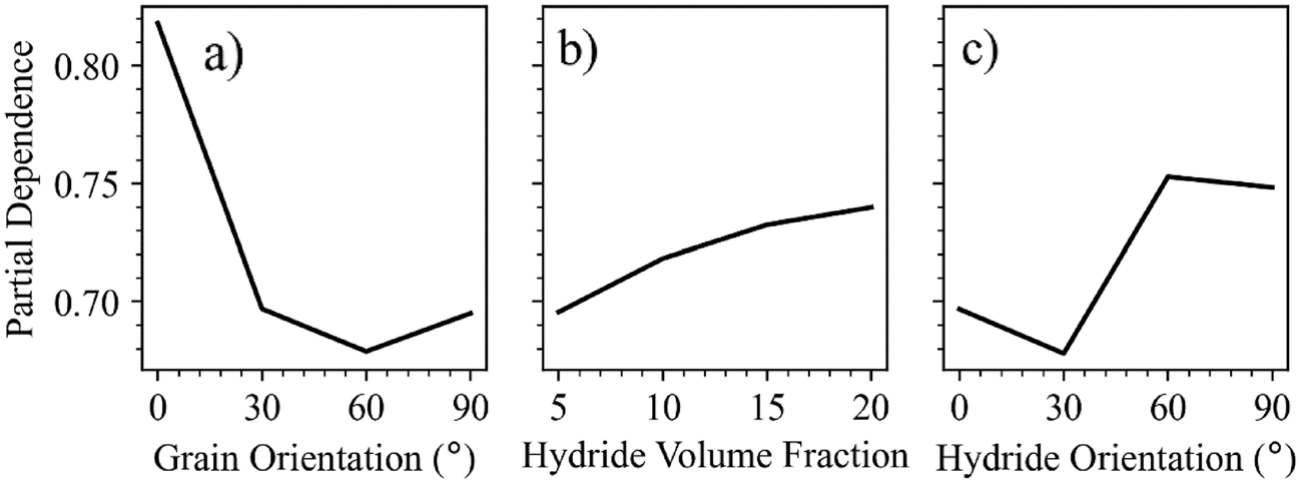
Partial dependence plots. The three highest ranked features normalized by fracture stress.

**Figure 6:**
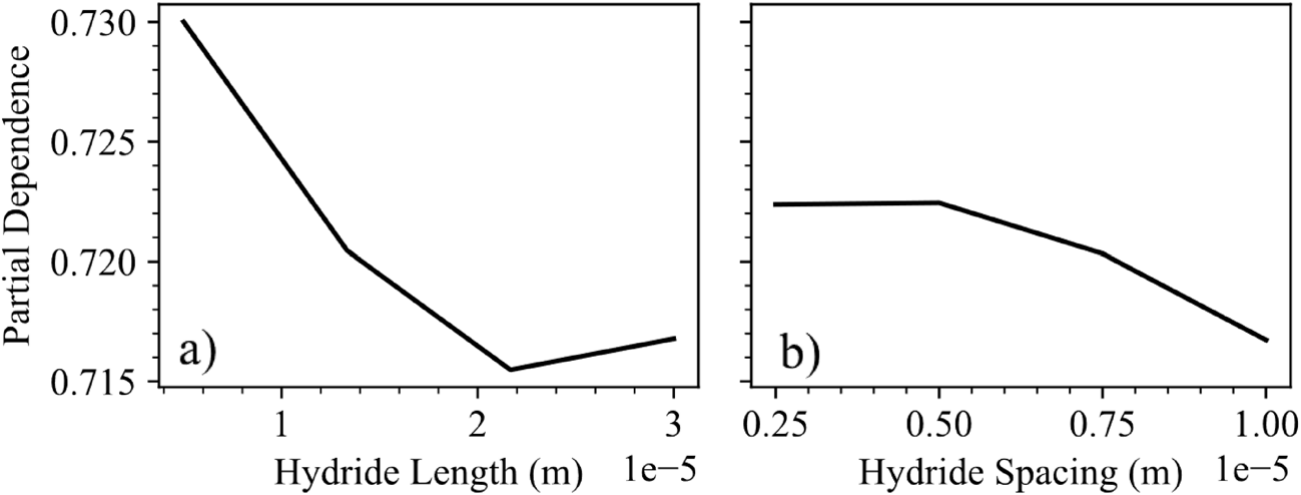
Partial dependence. Shown for hydride length and hydride spacing, showing minor variation.

A partial dependence study was also used to determine the how changing various features affects the critical fracture stress. The average model output at different constant values of the target feature was determined while varying the values for the other features. Figure 5 shows the individual dependencies for the three highest ranked parameters: grain orientation, hydride volume fraction, and hydride orientation. As grain orientation increases, the average fracture critical stress decreases until the material is at a 60° orientation and then increases again, showing the effects of preferred orientations where mobile dislocations in prismatic slip systems are most active and act to reduce the average fracture stress levels within the material. As hydride volume fraction increases, so too does the critical fracture stress, as expected because of the greater proportion of the material that consists of brittle hydride material. Finally, as hydride geometric orientation changes from radial to circumferential, the critical fracture stress levels tend to increase because of greater hydride tip interaction that occurs when the hydrides are oriented with the loading axis. This causes dislocation immobilization and increased fracture critical stress. Figure 6 shows the dependence for both the hydride length and the hydride spacing. It is important to note that the range of effect is much lower in these parameters when compared to the grain orientation, hydride volume fraction, and hydride orientation, which is in agreement with their reduced significance in the permutation importance study.

**Figure 7:**
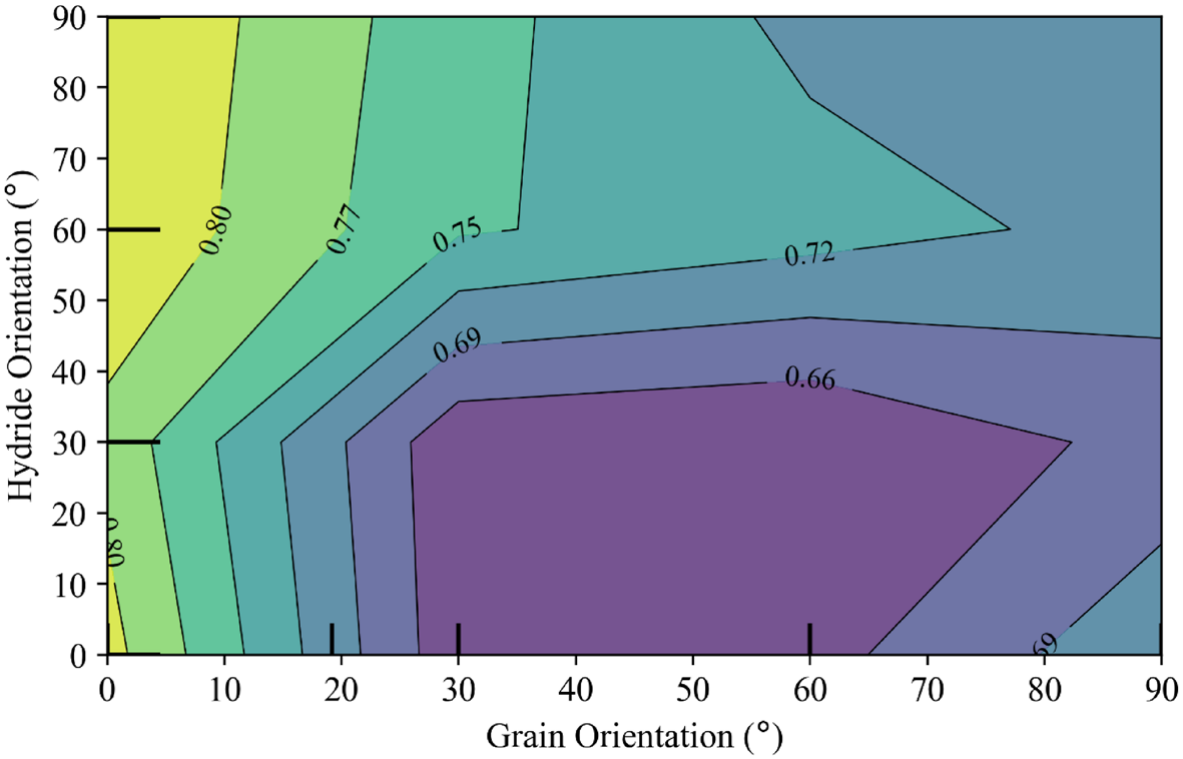
Partial dependence. The combined effects of grain orientation and hydride orientation are shown.

A contour plot of the partial dependence that incorporates both the grain orientation and the hydride orientation is shown in Fig. 7. This plot indicates that the highest fracture critical stress values tend to occur at radially hydrided materials with high misorientation with respect to the loading axis, and that the lowest values tend to occur with mixed hydrides and low misorientation with the loading axis.

**Figure 8:**
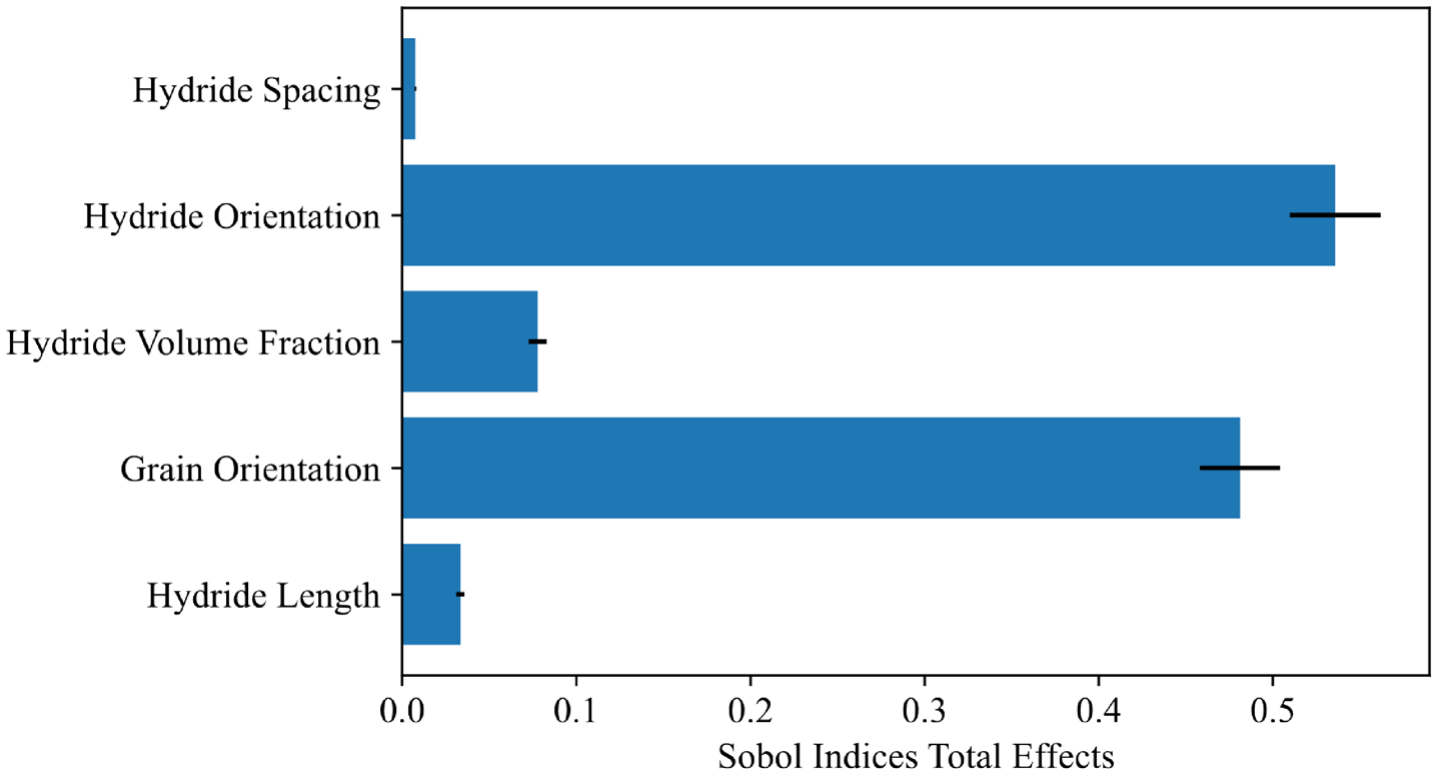
Sobol analysis for material fingerprint showing total effects**.** Calculated for the 3.25% nominal strain MLP model. 95% confidence intervals are indicated by black lines.

**Figure 9:**
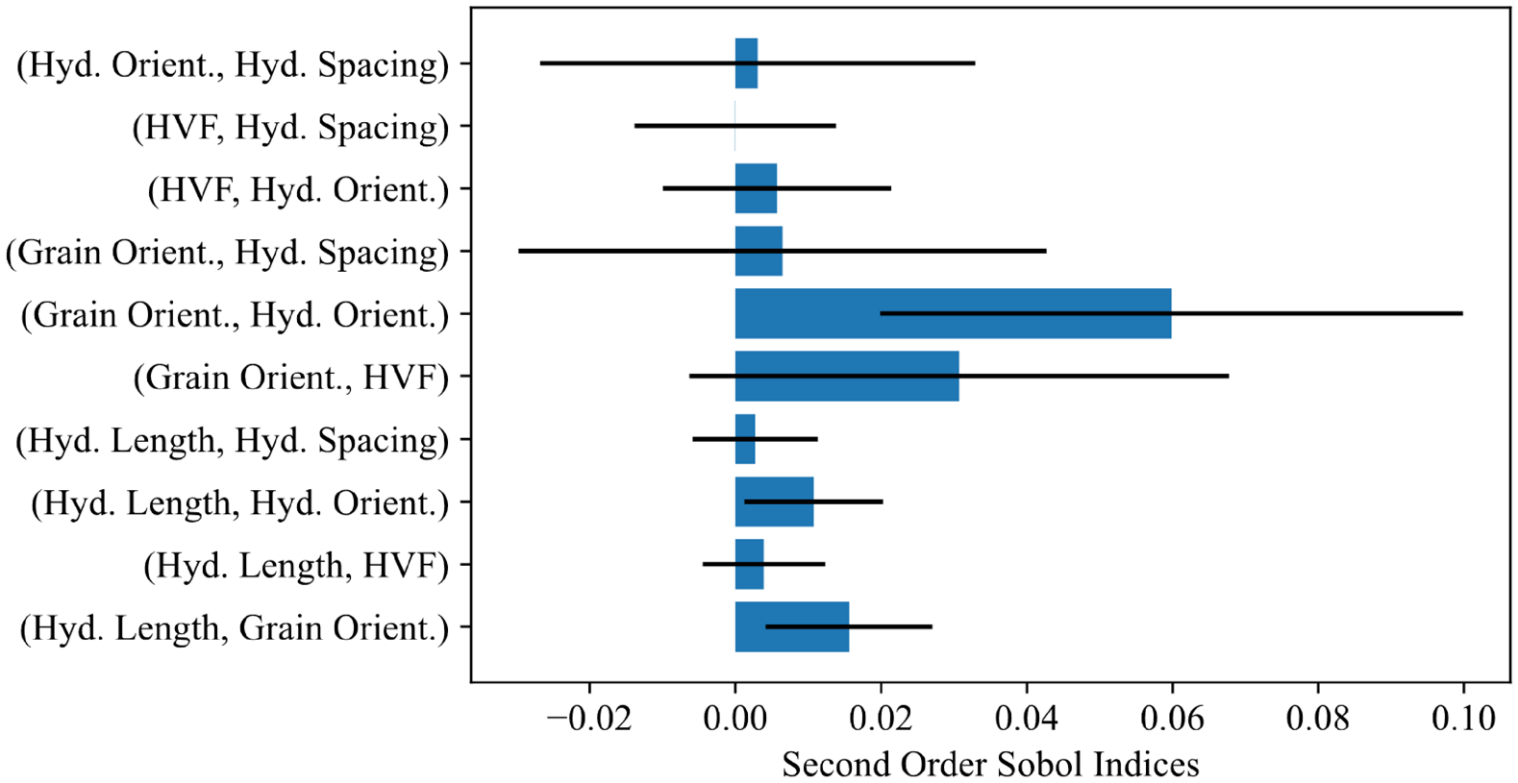
Sobol analysis for material fingerprint showing second order effects**.** Calculated for the 3.25% nominal strain MLP model. 95% confidence intervals are indicated by black lines.

Global sensitivity analysis was performed using the Sobol method on the 3.25% nominal strain MLP model. A Saltelli sampler was used with 5000 data points. The SAlib implementation in Python was used for sampling and analysis^26^. The results are shown in Figs. 8 and 9. They indicate a similar result to the previous parameter studies, with the highest indices attached to grain orientation, hydride orientation, and hydride volume fraction. The values of the second order indices are comparatively small, indicating that most of the model variance is attributed to single parameters. Hydride spacing was the least impactful parameter, as also shown in the feature importance study. If 0.05 is taken as the cutoff for an influential parameter^29^, then interactions with the grain orientation (texture) parameter are the only second order indices where the upper 95% confidence intervals fall within an influential range. The interactions observed between grain orientation and hydride volume fraction or hydride orientation are reasonable because material texture will impact dislocation accumulations within the material, thereby influencing the degree to which hydride orientation or volume fraction impact the material response.

### Significant factors influencing dislocation activity

**Figure 10:**
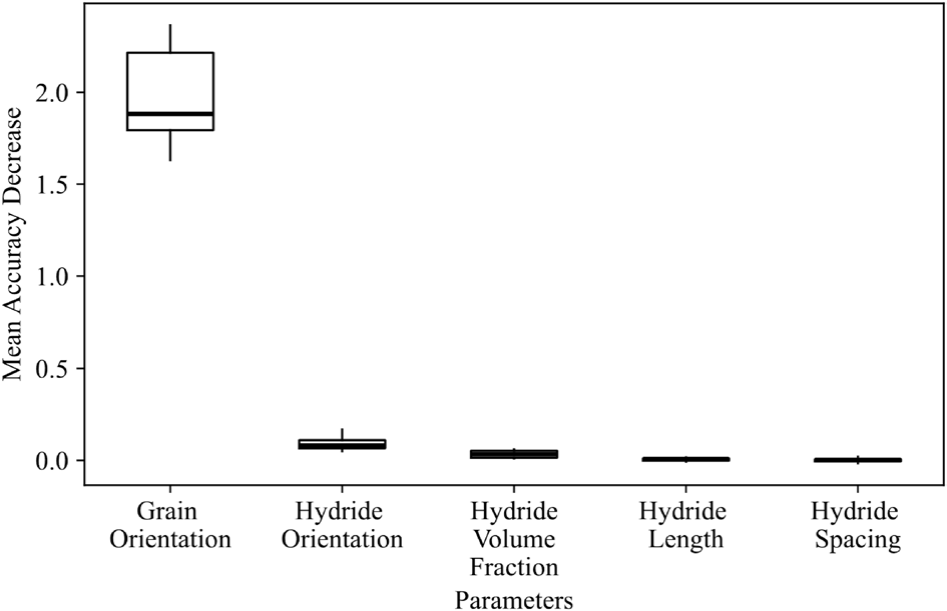
Feature importance for the maximum immobile density, ${\rho }_{im,max}$. Bar chart showing the dominance of material texture in predicting extremes of immobile dislocation density.

To further understand the role of dislocation-densities in affecting fracture, the immobilized dislocation density response was also modeled and analyzed to determine the relative importance of different features in the material fingerprint. The same method used to train a neural network for the critical fracture stress predictions was also used to train a model describing the maximum immobile dislocation density response, ${\rho }_{im,max}$, for all the modeled slip systems of the parent grain material. The training process resulted in a holdout test-set coefficient of determination of 0.98, indicating that the universal function approximator was able to capture the nonlinearities associated with the dislocation density evolution equations effectively. Figure 10 shows the results of a feature importance study on this model. Grain orientation is by far the most important parameter and hydride orientations do not seem to influence the maximum activation. This indicates that the orientation of the hydrides plays a role in the fracture critical stress predictions because of the geometric interaction between hydride orientations and trapped dislocations, and that the severity of the impact of dislocation accumulations on the critical fracture stress can be mainly influenced based on the hydride orientations. The material degradation caused by hydrides spanning the radius of the cladding tube, i.e., radial hydrides, is exacerbated by the accumulation of immobilized dislocation densities at preferential grain orientations.

## Conclusions

In this investigation, a database of deterministically FE modeled crystalline structures was used to find relationships between the proposed material fingerprints and fracture. Ordinary least squares, GPR, symbolic regression, random forest, and multilayer perceptron modeling techniques were evaluated for their performance in representing material critical fracture stress response. The parameters were evaluated to determine their contribution to the models, and to quantify their importance to the overall behavior with a focus on fracture stress.

Specifically:

1. Multilayer perceptrons (neural networks) had the best overall average performance, based on the coefficient of determination.
2. Grain and hydride orientations were predicted to be the feature most significant to the evolution of critical fracture stress level extremes.
3. Grain orientations statistically had the greatest impact on immobile dislocation density accumulation and activation along the preferred slip systems.
4. Hydride length and hydride spacing had relatively low significance in predicting critical fracture stress, which indicates that future studies might use a coarser representation of these parameters without loss of accuracy.

This investigation provides a detailed ML microstructural approach that can be utilized to understand and predict behavior in h.c.p. alloys with f.c.c. hydrides. The unique aspect of this approach is the coupling of deterministic FE models to ML, and it can be a new step in understanding and potentially controlling failure in multiphase materials through ML tools coupled to large strain plasticity and fracture methodologies, and this approach can be extended to random hydride orientations.

## Data Availability

All the relevant data for the manuscript is provided in https://github.com/tshasan-ncsu/fracture_nucleation.
